# Building a culture of safety in Australian residential aged care facilities: protocol for a longitudinal mixed methods research programme

**DOI:** 10.1136/bmjopen-2024-089293

**Published:** 2024-09-18

**Authors:** Kate Churruca, Jane Graham, Louise A Ellis, Johanna Westbrook, Nasir Wabe, Peter D Hibbert, Kristiana Ludlow, Rachel Urwin, Isabelle Meulenbroeks, Jey Thanigasalam, Ingerlise Svaleng, Jo-Ann Sardellis, Jeffrey Braithwaite

**Affiliations:** 1 Australian Institute of Health Innovation, Macquarie University, Sydney, New South Wales, Australia; 2 Calvary Health Care, Sydney, New South Wales, Australia; 3 Allied Health and Human Performance, University of South Australia, Adelaide, South Australia, Australia; 4 Centre for Health Services Research, Faculty of Medicine, The University of Queensland, Saint Lucia, Queensland, Australia

**Keywords:** Change management, Clinical governance, Health Services for the Aged, Quality in health care, Aged

## Abstract

**Abstract:**

**Introduction:**

The quality and safety of care within residential aged care facilities (RACFs) have been linked to their organisational culture. However, evidence for understanding and improving culture in this setting is limited. This research programme aims to validate a survey to measure organisational culture and determine the relationship of culture with safety and quality of care, then to evaluate an organisational culture change programme in Australian RACFs.

**Methods and analysis:**

This is a longitudinal mixed methods programme of research conducted across four studies in collaboration with a national aged care provider that cares for more than 5000 residents:

Study 1: Cross-sectional staff survey of organisational culture in >50 RACFs with concurrent collection of data on quality and safety of care, and staff outcomes, to explore their associations with culture.

Study 2: Ethnographic fieldwork in eight RACFs sampled to achieve maximum variation. Data from interviews, observations and documents will be analysed to identify the underlying assumptions and how cultural assumptions influence the enactment of safety and quality.

Study 3: Evaluation of the implementation of the Speak Up for Safety culture change programme, focusing on its contextualisation for RACFs, implementation determinants and outcomes. Data will be collected through semistructured interviews, complimented with secondary data from program training and feedback system usage.

Study 4: Evaluation of the effectiveness of the culture change programme using baseline data from study 1 and a follow-up survey of organisational culture postimplementation to assess changes in organisational culture and staff behaviour.

**Ethics and dissemination:**

The study has received approval from the Macquarie University Human Research Ethics Committee. Informed consent will be sought from all participants. Findings will be disseminated through journal articles, conference presentations and reports to the collaborating provider and RACFs. Survey data will be deposited into a data repository for use by others working on related research.

**STRENGTHS AND LIMITATIONS OF THIS STUDY**

- Strong collaboration with a large national aged care provider.
- Mixed method research programme with multiple data collection methods used to understand organisational culture and culture change in Australian residential aged care.
- Use of an implementation determinant framework, the Consolidated Framework for Implementation Research, to identify factors influencing the implementation of the culture change programme and effects on implementation outcomes.
- It may not be possible to include a control group in evaluating culture change programme effectiveness.

## Introduction

 Australia’s aged care sector has faced numerous inquiries into the quality of care over the last decade (eg, The Oakden Report,^1^). The most prominent example, the 2018–2021 Royal Commission into Aged Care Quality and Safety,^2^ estimated that one in three residents in residential aged care facilities (RACFs) experienced substandard care. The Australian CareTrack Aged study, published in 2024, found that only just over half (53.2%) of care delivered in RACFs was in line with evidence.^3^ Other investigations have highlighted the overuse of physical restraints and antipsychotic medications,^4^ inappropriate or missed care,^5 6^ understaffing and other staffing issues including high turnover, and poor training and communication.^7^ In public inquiries, inadequate quality of care has been repeatedly linked to issues in the organisational culture of RACFs and providers.^1 8 9^

### Background

**Figure 1:**
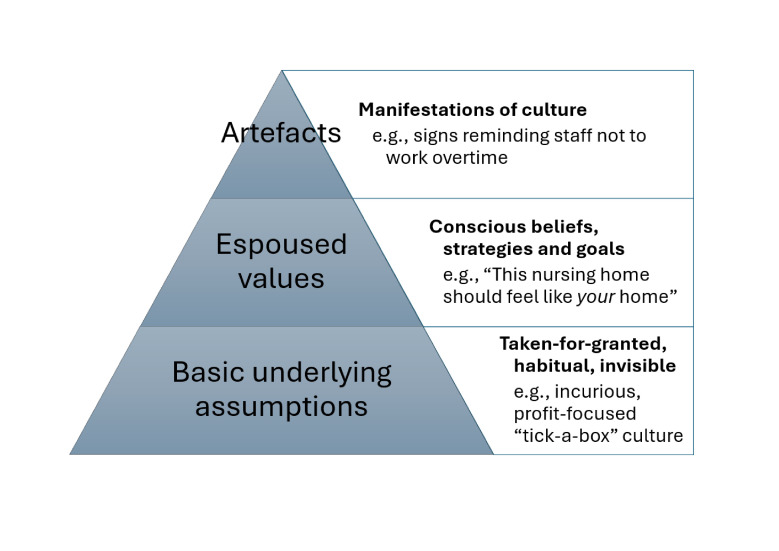
Three layers of organisational culture with examples from residential aged care.

Often colloquially described as ‘the way we do things around here’, organisational culture can be defined as a pattern of assumptions that is shared among those working within an organisation and influences their norms, attitudes, feelings, beliefs and behaviours.^10^ In a widely utilised theoretical framework,^11 12^ Schein^10^ delineates three levels of organisational culture, with cultural artefacts being the most conspicuous layer; these include workplace rituals, behaviours, structures, dress codes and the physical environment. Espoused values guide what is important within an organisation and how work gets done and comprise the second, less readily observable, but still accessible layer. These values can be seen in mission statements, policies and official organisational communication. Finally, at the heart of an organisation’s culture is its basic underlying assumptions, tacit or even unconscious expectations and values that influence how organisational members interpret events, make decisions and interact with one another. Figure 1 summarises these layers of organisational culture with examples drawn from aged care.

Across healthcare settings, positive organisational cultures, characterised by features like effective teamwork, good leadership and open communication, are associated with better patient outcomes, including reduced falls, mortality and healthcare-acquired infections, and increased patient satisfaction.^13^ Compared with other healthcare settings, RACFs, also known as long-term care, nursing homes or care homes, provide a more holistic care environment for individuals unable to live independently, including medical, social, emotional and spiritual care, and support with activities of daily living.^6^ In this setting, studies have found that a stronger and more positive safety culture is correlated with fewer system deficiencies and complaints, higher quality ratings^14^ and the likelihood of people delivering person-centred care,^15^ and that culture contributes to the levels of prescribing psychotropic medicines.^16^ Our integrative review of 92 studies internationally on organisational culture in residential aged care also found evidence to suggest a relationship between culture and staff-level outcomes (eg, job satisfaction) and clinical care processes (eg, use of psychotropic medicine); however, research demonstrating an association between culture and clinical outcomes (eg, pressure ulcers) was more equivocal.^11^

The most widely studied facet of organisational culture in healthcare is safety culture.^11 12 17 18^ It is considered fundamental to improving the safety and quality of healthcare organisations,^19^,^21^ with its assessment increasingly used as an indicator of quality for regulation or accreditation. In Australia, national safety and quality standards require acute care managers to develop and monitor their safety culture.^22^ Likewise, in the quality standards introduced for aged care in 2019, the governing body of an RACF is expected ‘to promote a culture of safety and quality, and to include this in the organisation’s governance system’.^23^

Surveys are useful for understanding safety culture, having advantages in their efficiency, in providing data suitable for comparison and monitoring over time, and diagnosing discrete issues (eg, handover).^24 25^ Safety culture surveys have primarily been conducted in acute care settings,^13^ with very few studies on safety culture undertaken in Australian RACFs.^12^ Moreover, measurement tools for safety culture in aged care, such as the Nursing Home Survey on Patient Safety Culture, are informed by their hospital counterparts.^26^ While they offer good coverage of clinical care processes, they arguably do not focus on all aspects of culture that may be important to providing quality care in this setting. RACFs differ from hospitals in that >70% of staff are personal care workers without clinical expertise,^27^ and facilities are long-term homes for residents who require a more holistic approach to care, contrasted with an episodic approach in acute settings.^6 12^ Organisational norms and values related to person-centredness are particularly important in RACFs^28^ and are already known to be associated with safety culture.^15^ Likewise, recent trends, in safety culture measurement that recognise the roles of consumers, patients and their families in safety,^18^ are even more pertinent in residential aged care where residents may be regularly visited by family members acting as informal caregivers.

A fit-for-purpose and validated survey can provide a snapshot of the relative strengths and areas for improvement in the culture of Australian RACFs, facilitate internal monitoring, identify relationships with other organisational characteristics and support the development and evaluation of interventions to improve culture. However, returning to Schein’s framework of culture, surveys do not surface the most deep-seated facet of culture, its basic underlying assumptions. This requires the use of interpretive methods such as ethnography,^10^ which are rarely applied in this setting to understand organisational culture.^11 12^ With this in mind, a mixed methods approach that leverages quantitative breadth with qualitative depth is considered most appropriate for building the knowledge base on how organisational culture influences care and how culture change can be achieved.

Such work is urgently needed, as policy guidance and empirical evidence to support culture change are limited.^9^ Our integrative review of organisational culture in RACFs found only five interventional studies that explicitly sought to improve culture.^11^ These generally lacked details on how culture was targeted with some not using study designs capable of demonstrating effectiveness, and none considering implementation issues. To address these gaps, this research programme aims to validate a survey to measure organisational culture and determine how culture affects safety and quality of care (aim 1) and then to evaluate an organisational culture change intervention in Australian RACFs (aim 2).

## Methods and analysis

**Figure 2:**
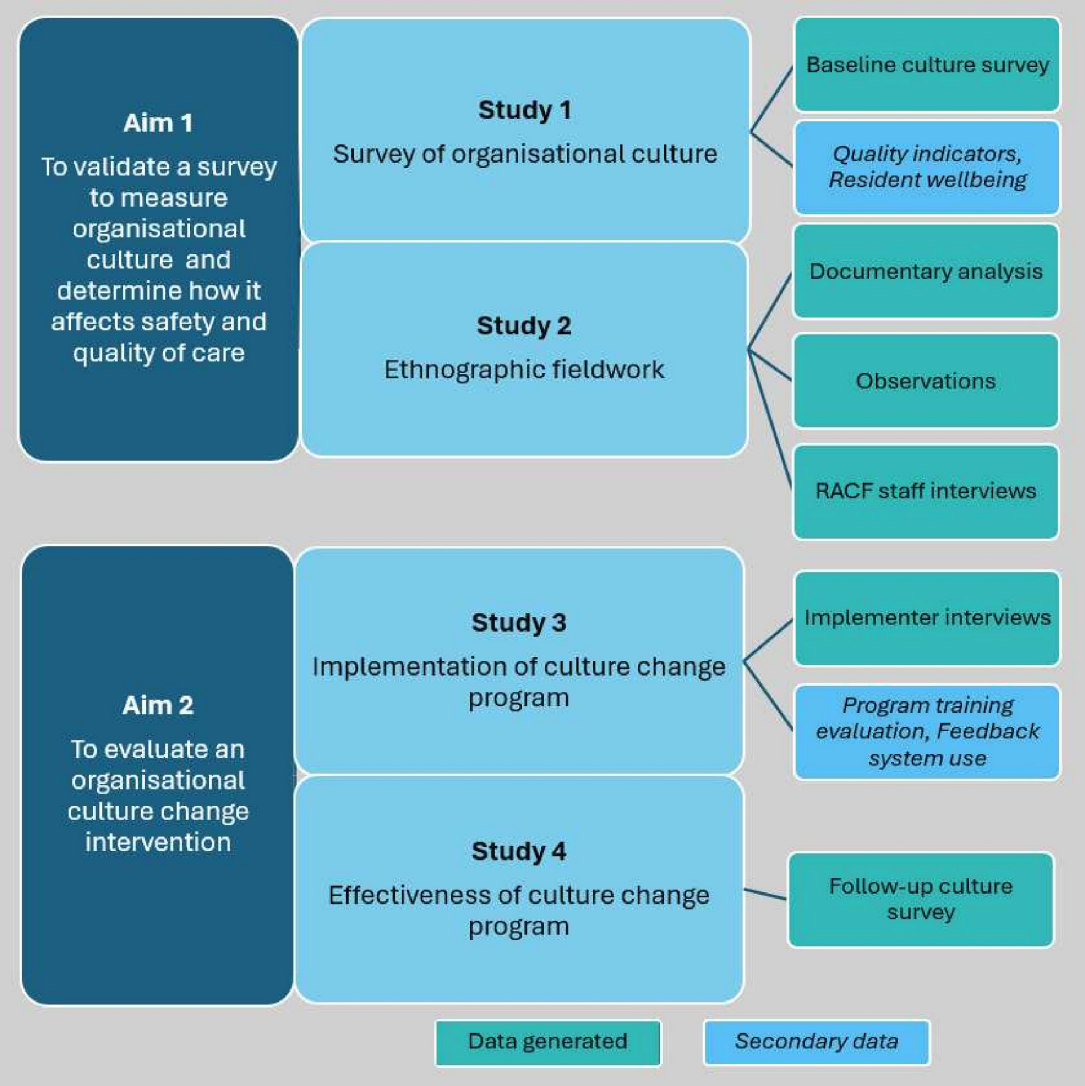
Summary of research programme. RACF, residential aged care facility.

We propose a longitudinal mixed method research programme involving the assessment of organisational culture over time and the evaluation of a culture change programme in Australian RACFs. Over 4 years, qualitative and quantitative data will be collected in four studies through surveys; ethnographic fieldwork with observations, interviews and documentary analysis; use of secondary clinical and staff-reported data; and interviews with key stakeholders in the implementation of the culture change programme. Figure 2 summarises the overarching research programme.

### Research collaboration and setting

This research is being conducted in collaboration with a private, not-for-profit provider of health and aged care services (the provider). The provider operates over 50 RACFs caring for more than 5000 residents at any point in time. Its facilities are located across Australia’s east, including in the Australian Capital Territory, New South Wales, Queensland, South Australia, Tasmania and Victoria, and in metropolitan areas, regional centres, and large, medium and small rural towns. Facilities range in size from 30 to more than 200 resident beds.

### Study 1: Survey of organisational culture in RACFs

Study 1 will be used to quantitatively address aim 1, delineating the effects of different aspects of organisational culture on safety and quality of care, and staff outcomes, and validating a multidimensional survey of organisational culture for Australian RACFs. All staff employed by the provider and working within participating RACFs will be eligible to take part, including nurses, personal care workers, management, administrative and support staff. A sample size of approximately 2000 will be targeted, assuming a 50% response rate and with an average of 75 staff working in each facility.^27^

We will design a survey to assess organisational culture and examine its relationship with a range of staff outcomes. The tool will be based on the Nursing Home Survey on Patient Safety Culture by the Agency for Healthcare Research and Quality in the USA,^29^ which contains 12 safety culture composites including teamwork, communication openness and organisational learning. Despite widespread use internationally,^11^ it has not been adapted and validated for the Australian context. Items will also be added to evaluate other important aspects of culture within residential aged care, including items on providing person-centred care,^30^ and family member involvement in safe care.^31^ In addition to these dimensions of culture, additional scales measured in the survey will include frequency of experience of common unprofessional behaviours from coworkers,^32^ staff burnout,^33 34^ intention to leave and staff demographics. The survey will be anonymous, and responses to questions voluntary.

In 2024–2025, staff working in participating facilities will be invited to take part in the survey online through REDCap, or on a paper form. A designated point of contact working within each facility (eg, facility manager, senior administrative staff) will send out email invitations to the online survey and support hardcopy administration. Using multiple administration methods increases the chances of a high response rate^35^ and overcomes the issue that many aged care staff may not have easy access to a computer during their shift. The survey will be further supported by executive endorsement through the provider’s internal communications channels. These communications will include regular updates and promotions, aiming to drive engagement in the research by connecting it with the overall organisational strategy and mission, roll out of the culture change programme and with recognition of individuals and teams involved in the work.

To examine the association of culture with safety and quality of care, quality indicator and resident experience data will be accessed from the Australian Government’s GEN Aged Care Data website.^36^ These indicators are mandatory for RACFs to report and are compiled and publicly released in the Star Rating Quarterly Extract.^37^ Quality indicator data are aggregated to a facility-level and expressed as the percentage of residents experiencing a range of issues in five areas of care: (1) pressure injuries, (2) physical restraint, (3) unplanned weight loss, (4) falls and major injury and (5) medication management. Resident experience ratings are collected annually and published in the same extract. The most recent data release directly following the survey will be used for each facility.

Survey data will be analysed using SPSS and AMOS.^38 39^ To first validate the survey, the psychometric properties of the organisational culture dimensions will be assessed via exploratory and confirmatory factor analysis through inspection of factor loadings, goodness of fit indices and model fit. Internal consistency reliability will be tested through Cronbach’s alpha. Structural equation modelling, and where possible multilevel modelling, will then be used to examine relationships among variables, including the relationship between various aspects of organisational culture and staff level outcomes assessed in the culture survey (eg, burnout, intention to leave), and the degree to which facility-level culture predicts indicators of aged care quality.

### Study 2: Ethnographic fieldwork

In 2025–2026, eight sites will be selected for ethnographic fieldwork based on facility characteristics and the results of the first round of the survey (study 1) using maximum variation sampling.^40^ This will ensure coverage of a range of facilities in terms of size, location and scoring on the dimensions of organisational culture. During fieldwork, an experienced qualitative researcher will spend approximately 30–40 hours in each facility spread over a range of different shifts.^41 42^ The researcher will conduct general observations, direct observations of individual staff members and semistructured interviews, complimented by documentary analysis. They will use Schein’s^10^ three-layer conceptualisation of culture as a methodological framework to understand in depth *how* aspects of organisational culture within each facility influence the safety and quality of care, further addressing aim 1.

Fieldwork will commence with general observations that focus on recording information about the physical environment of the facility (eg, signs, notices, dress codes, layout, artwork) and public team interactions (eg, meetings). Documents will be sourced from RACFs, including mission statements; policies, rules and codes of conduct; provider and management emails distributed to all staff; and organisational charts. This information will be used to develop an understanding of the broader context of the facility, and particularly cultural artefacts. We will then conduct direct observations of individual staff members (n=8–10 from each facility), sampling from a variety of roles (eg, leadership, registered nurses, direct care workers, allied health staff) and prioritising those who have been employed at the facility for longer. These sessions will involve shadowing participants during their workday. The researcher will take fieldnotes documenting behaviours and events observed, questions raised and emerging interpretations and may ask the observed staff member clarifying questions where safe and appropriate to do so. These sessions are intended to surface further cultural artefacts and staff’s espoused beliefs, but may also, with corroboration from other sources and data collected as part of this study, elucidate some of the basic underlying assumptions of the organisation.

Staff involved in direct observations will then take part in a semistructured interview, in which the researcher will ask them about norms and behaviours related to quality and safety of care in the facility, test emerging interpretations about the organisation’s underlying assumptions and request further clarification on observations. Residents and family members (n=3–5 from each facility) will also be asked to participate in interviews as key informants on culture. Given the long-term nature of care, this group will have accumulated insights into the organisational culture and its role in care delivery, and yet their perspectives have rarely been sought in studies on culture.^11 12^

Audio-recorded interviews will be transcribed, and then transcripts, fieldnotes and organisational documents will be deidentified at individual-level and facility level and imported into NVivo for thematic coding and analysis.^43^,^45^ This will involve inductive coding to classify manifest meaning in the data; codes will then be refined in multiple iterative rounds and linked together to develop themes that convey broader, recurrent patterns and latent aspects of meaning (eg, cultural assumptions). Coded data will be triangulated between different sources and methods of collection and compared between facilities to unravel common and unique elements of culture. Findings will be summarised through ‘thick description’, an analytical process used in ethnography to create a detailed, explanatory interpretation of behaviours with reference to the context, human emotions and social connection.^46 47^

### Study 3: Implementation of a safety culture change programme in residential aged care

The provider has implemented a multicomponent programme aimed at improving safety culture in their private and public hospitals and has plans to roll this out across their other services, including residential aged care in 2024–2027. The programme in full is called the Speak Up for Safety (SUFS) programme; it focuses on fostering a culture in which all staff are responsible for maintaining safety and feel able to speak up about issues and behaviours that might lead to harm to patients, residents or staff.^48^ The SUFS programme components include a common language for raising and escalating safety concerns and a model to promote professional accountability and reduce unprofessional or unsafe staff behaviour. The latter part is based on the Vanderbilt approach to promoting professional accountability,^49 50^ which has been implemented across a range of hospitals, including in Australia.^3251^,^54^ However, studies investigating the effectiveness and implementation of culture change programmes, such as those focused on improving safety culture and professional accountability, are lacking in aged care. Table 1 summarises the core components of the programme as they have been implemented in hospitals.

**Table 1:** Core components of the Speak Up for Safety culture change program

Programme components	Explanation
Speaking Up for Safety (Safety C.O.D.E.)	A standardised, graded assertiveness approach to communicating about safety concerns in a professional way that maintains respect for a coworker’s skills and expertise. It encourages staff to proactively identify potential risks to patient and staff safety and speak up in the moment to mitigate this. These components are trademarked to the Cognitive Institute^48^ and The Medical Protection Society and rolled out by the provider under a service and licence agreement.
Model for promoting professional accountability	A tiered model with graduated interventions for addressing unprofessional behaviours that undermine safety. It begins with early, informal and non-punitive peer feedback on behaviour. The feedback is collected through the feedback system.
Feedback system	A secure online system in which staff can confidentially submit feedback on their coworkers’ behaviours that either promote or undermine safety, for use if there is no imminent risk of harm and they are unable to speak up in the moment, or to a manager.
Triage team	Trained staff who review submissions to the online system, evaluate their contents and where appropriate forward them along for delivery.
Leader/peer messenger	A member of staff who is trained to deliver feedback related to unprofessional behaviour in a non-judgemental way that encourages the receiving staff member to reflect on, and ideally change, their behaviour.

Programme implementation	Explanation
Phased training and roll out	Elements of the programme are introduced in a phased way, beginning with training in the Safety C.O.D.E. provided to all staff. Leaders/peer messengers are also trained in having difficult conversations with their staff. Once approximately 80% of staff have received Safety C.O.D.E training, the feedback system is rolled out.
Train-the-trainer (Safety C.O.D.E related)	To foster buy-in and programme sustainability, the programme is intended for roll out via members of the organisation who are trained by the programme owner.

In recognition of the difference in the environment of residential aged care, compared with the hospitals where professional accountability and safety culture programmes have primarily been implemented, the provider has established a working party to facilitate the roll out of the programme. The group includes facility, regional and national managers for aged care, programme experts and specialists in learning and development. The provider’s plans for the implementation are still emergent but will include a staged roll out across its large number of RACFs.

This study addresses aim 2 by developing an understanding of the process by which the culture change programme is adapted for, and implemented in, residential aged care. Semistructured interviews will be conducted with a range of key stakeholders, including staff within facilities; aged care managers at sites, regionally and nationally; leads for clinical governance; and covering off those in key programme roles (eg, conducting training, peer messengers, triage team member). We anticipate approximately that 50 interviewees, with a minimum of two to three participants from each group involved in programme implementation, will provide sufficient coverage of the topic, based on prior experience. Interviews will take place throughout the roll out of the programme (2024–2027) and will be conducted either in person, or via videoconferencing. During interviews, participants will be asked questions aimed at identifying implementation determinants as conceptualised by the Consolidated Framework for Implementation Research (CFIR).^55 56^ A member of the research team with expertise in implementation science will also attend working party meetings, making notes on the process for adapting and implementing the programme in RACFs. These notes will supplement interviews and contribute further insights into decision-making around the process and strategies for implementation. Secondary data generated by the provider will also be sought, including programme training evaluations and data on use of the online feedback system.

Audio-recorded interviews will be transcribed, deidentified and imported into NVivo for analysis; meeting notes and secondary data will also be imported into NVivo.^45^ Content analysis will then be conducted,^57^ which will involve deductive coding to capture domains and constructs of the CFIR,^55 56^ implementation strategies^58 59^ and implementation outcomes.^60^ CFIR-coded extracts will then be evaluated to examine if the specific construct had a positive (enabler), negative (barrier) or mixed impact on the implementation of the programme at a facility or provider level. Identified barriers, enablers and mixed determinants will be interpreted with reference to the implementation strategies used, and in relation to implementation outcomes including acceptability, appropriateness, adoption, feasibility, fidelity and penetration.^60^

### Study 4: Effectiveness of a safety culture change programme in residential aged care

As part of aim 2, to evaluate the effectiveness of the culture change programme, survey data collected for study 1 will be used as a baseline measure of culture for each facility. The organisational culture survey will then be repeated at least once 12–18 months later and following the implementation of the culture change programme in each RACF (2026–2027). Together, these data will be used to evaluate the effectiveness of this programme, focusing on changes in organisational culture scores and the frequency of experienced unprofessional behaviours (main outcome measures). In addition, aggregated facility-level data collected by the provider will be used to investigate the impacts of the programme on secondary outcomes including staff engagement and turnover.

Comparisons of facility means for preimplementation and postimplementation of the intervention will be carried out on the main and secondary outcome measures. Given the plan for a phased programme roll out across RACFs, it may be possible to conduct a pre–post analysis (eg, analysis of variance (ANOVA)) with a control group, in the event a sufficient number and range of facilities have not yet received the programme. Otherwise, paired-samples t tests will be used to assess change following programme implementation.

### Patient and public involvement

Patients and members of the public were not directly involved in the design of this research programme. However, much of the work that has highlighted the need for this research, such as the Royal Commission into Aged Care Quality and Safety has been strongly informed by consumers’ concerns, which were a major part of the enquiry. Aged care residents and their families will contribute actively to study 2 as key informants on RACF culture, and secondary data on resident well-being will be used in study 1. Given the staged nature of the research, and the research teams involvement in the working party, insights from residents and their family provided during data collection can be fed back to the provider and may be used to refine the roll out of the culture change programme. The research institute conducting this programme of work also has a consumer panel whose expertise will be drawn on throughout the project to provide feedback on analysis, and the dissemination of findings.

## Ethics and dissemination

### Ethics

This study has received ethics approval from the Medicine and Health Sciences Subcommittee of the Macquarie University Human Research Ethics Committee (ref no. 520221260244174). All participants will opt in and provide voluntary informed consent for their participation in the research. Specifically for the ethnographic fieldwork in study 2, multiple levels of consent will be sought beginning with broad consent from facility managers, individual consent from participants being shadowed or interviewed, and in situ consent from any person this individual comes into contact with during observations. Aside from the inconvenience of filling in a survey, or the discomfort of being observed, there is a low probability that some staff members will experience distress at being asked questions about their facility’s culture and their potential experiences of burnout. Respondents will be provided with contact information for counselling and support services at the end of the survey if participation raises any issues. Facilities involved in this research may view the results of this research as a potential risk to their reputation. To negate this, data will be deidentified at a facility level.

### Dissemination

Study findings will be disseminated in peer-reviewed articles, and conference presentations in the form of aggregated data and illustrative participant quotes. Findings will be fed back to the provider throughout the research programme in reports, presentations and informal communication. Facility-level reports to participating RACFs may be provided if sufficient survey responses are received (ie, >10 and minimum 30% of staff), and scores may be compared with group means. Organisational culture survey data collected for studies 1 and 4 will be deposited into a data repository and made available to researchers working on related projects and with appropriate ethics approval.
