# Supplementary material for: Building a culture of safety in Australian residential aged care facilities: protocol for a longitudinal mixed methods research programme
Source: BMJ Open. 2024 Sep 18;14(9):e089293. doi: 10.1136/bmjopen-2024-089293 (PMC11429349; doi:10.1136/bmjopen-2024-089293)
Supplement: Supplementary file 1 [file bmjopen-2024-089293.reviewer_comments.pdf]

PEER REVIEW HISTORY

BMJ Open publishes all reviews undertaken for accepted manuscripts. Reviewers are asked to complete a checklist review form (**http://bmjopen.bmj.com/site/about/resources/checklist.pdf**) and are provided with free text boxes to elaborate on their assessment. These free text comments are reproduced below.

ARTICLE DETAILS

**TITLE (PROVISIONAL)**	Building a culture of safety in Australian residential aged care facilities: Protocol for a longitudinal mixed methods research program
**AUTHORS**	Churruca, Kate; Graham, Jane; Ellis, Louise A.; Westbrook, Johanna; Wabe, Nasir; Hibbert, Peter; Ludlow, Kristiana; Urwin, Rachel; Meulenbroeks, Isabelle; Thanigasalam, Jey; Svaleng, Ingerlise; Sardellis, Jo-Ann; Braithwaite, Jeffrey

This article was not externally reviewed at BMJ Open. Protocols that have been independently assessed prior to submission to BMJ Open are usually fast-tracked to publication on the grounds that further substantial changes will not be possible. This independent assessment will usually be external, independent review for both a substantial grant award from a non-commercial or government funder and ethics approval.
